# Molecular Networking, Docking, and Biological Evaluation of Licarin A from *Myristica fragrans* as a Potential Cancer Chemopreventive Agent

**DOI:** 10.3390/molecules29204919

**Published:** 2024-10-17

**Authors:** Peter J. Blanco Carcache, Ines Y. Castro-Dionicio, Nathan P. Mirtallo Ezzone, Eric D. Salinas-Arrellano, Joshua Bahar, Steven K. Clinton, A. Douglas Kinghorn

**Affiliations:** 1 The Ohio State University Comprehensive Cancer Center, Columbus, OH 43210, USA; blancocarcache.1@buckeyemail.osu.edu; 2 Division of Medicinal Chemistry and Pharmacognosy, College of Pharmacy, The Ohio State University, Columbus, OH 43210, USA; castro.206@osu.edu (I.Y.C.-D.); ezzone.4@buckeyemail.osu.edu (N.P.M.E.); danisalinass@hotmail.com (E.D.S.-A.); joshuabahar@brandeis.edu (J.B.); 3 Division of Medical Oncology, College of Medicine, The Ohio State University, Columbus, OH 43210, USA

**Keywords:** cancer chemoprevention, licarin A, molecular networking, docking, zebrafish, lack of toxicity

## Abstract

Currently, clinically available cancer chemopreventive drug options are limited to mostly tamoxifen and its derivatives, such as raloxifene, and approved specifically for breast cancer. Thus, the availability of chemopreventive drug molecules for other types of malignant cancers would be desirable. In previous reports, the arils of *Myristica fragrans* (mace) have been found to exhibit cancer chemopreventive activity. Therefore, the purpose of the present study was to identify a natural product from this species with potential chemopreventive activity guided by chemoinformatic sample analysis via Global Natural Products Social (GNPS) molecular networking and molecular docking. The neolignan licarin A (**1**) was identified as a potential chemopreventive constituent, and subsequently submitted to several in vitro bioassays and a zebrafish toxicity evaluation. In this work, **1** afforded superior phosphoNF-*κ*Bp65 phosphorylation activity in DU-145 prostate cancer cells compared to isoliquiritigenin (**2**), which was used as a natural product chemopreventive control. Both **1** and **2** showed a longer-lasting reduction in cellular stress in a cell oxidative stress real-time dose–response assay than the positive control using Hepa1c1c7 mouse hepatoma cells. In addition, **1** displayed similar activities to **2**, while also being less toxic to zebrafish (*Danio rerio*) than both this chalcone and the clinically used chemopreventive drug tamoxifen.

## 1. Introduction

Cancer chemopreventive drug agents in the clinic are approved currently only for breast cancer. The present options are limited to tamoxifen and its derivatives as well as the aromatase inhibitor exemestane, but these have various side effects that have not been completely overcome [1]. Other malignancies, such as colon, breast, prostate, and lung cancer, still require reliable chemopreventive options [2]. Therefore, the purpose of the present study was to identify a natural product compound with potential cancer chemopreventive activity from the spice mace (*Myristica fragrans* Houtt., Myristaceae). Many natural products of plant origin have shown a potential for use as cancer chemopreventive agents [3,4,5,6].

The arils of *Myristica fragrans* (mace) are used as a traditional spice for culinary purposes and medicinally in various systems of traditional medicine. Among the known bioactive constituents of this species are various phenylpropanoids and neolignans [7]. A chemopreventive effect of mace, when administered in the diet, was described using a mouse skin model, with dimethylbenz[*a*]anthracene (DMBA) as a carcinogen and croton oil as a tumor-promoting agent [8]. Garam masala is a food seasoning mixture comprised of nine spices inclusive of mace, and an aqueous extract showed cancer chemopreventive activity in murine transplacental and translactational models that were induced with DMBA [9]. Myristicin, a phenylpropanoid known to occur in the volatile oil of *M. fragrans* [10], was shown as a constituent of the leaf oil parsley (*Petroselenum sativus*) to inhibit benzo[*a*]pyrene-induced tumorigenesis in female mice [11]. In addition, this compound acted as an inducer of the detoxifying enzyme glutathione *S*-transferase and down-regulated genes involved in DNA damage [10,11,12]. Another compound from *M. fragrans*, licarin A, has been documented as having various biological effects, inclusive of antimycobacterial [13], antischistosomal [14], and DPPH radical scavenging [15] activities, as well as the ability to promote ABCG2 gene expression [16].

In the present study, preliminary evidence is presented that licarin A (**1**), as a previously known constituent of mace [15,17], seems worthy of further investigation for its potential cancer chemopreventive activity, with limited toxicity. This was achieved by employing molecular networking and molecular docking processes to identify a compound that could be later validated in vitro and in vivo as part of an innovative approach to cancer chemoprevention drug discovery. Chemoinformatic analysis was performed using the Global Natural Products Social (GNPS) molecular networking of mace and a UHPLC-ESI-Q Exactive system hyphenated with ESI-MS/MS in tandem, employing SIRIUS computational analysis across various databases and CSI:Fingerprint ID for structural annotation [18,19]. Compounds identified within the molecular network correlated to **1** in region A (compounds **3**–**6**) or possessing a similar chemical structure within the neighboring region B (compounds **7**–**10**) were chosen for in silico molecular docking analysis.

**Figure 1:**
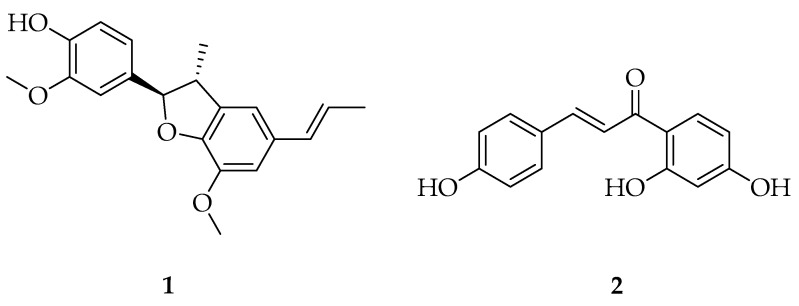
Structures of licarin A (**1**) and isoliquiritigenin (**2**).

The binding affinity and interactions with NF-*κ*Bp65 and PARP-1 were determined for all these compounds and compared to **1** to further explore potential cancer chemopreventive compounds from *M. fragrans* in future work. Isoliquiritigenin (**2**) has been demonstrated previously as a natural product chemopreventive agent [6,20]. However, this compound is limited in its possible drug application due to its toxicity [21]. Accordingly, **1** and **2** (Figure 1) were tested in in vitro cell viability, phosphoNF-*κ*Bp65 cytosolic translocation, and cell oxidative stress bioassays to compare their mechanisms and duration of activity by utilizing DU-145 human prostate cancer cells and Hepa1c1c7 mouse hepatoma cancer cells. Finally, as an initial screen of toxicity, **1** was administered to zebrafish (*Danio rerio*) at a high dose to evaluate its potential safety in an animal model, in comparison to the clinical chemopreventive drug tamoxifen and **2**.

## 2. Results

### 2.1. Applications of Molecular Networking of Myristica fragrans to Target the Presence of 1 for Isolation and Characterization

**Figure 2:**
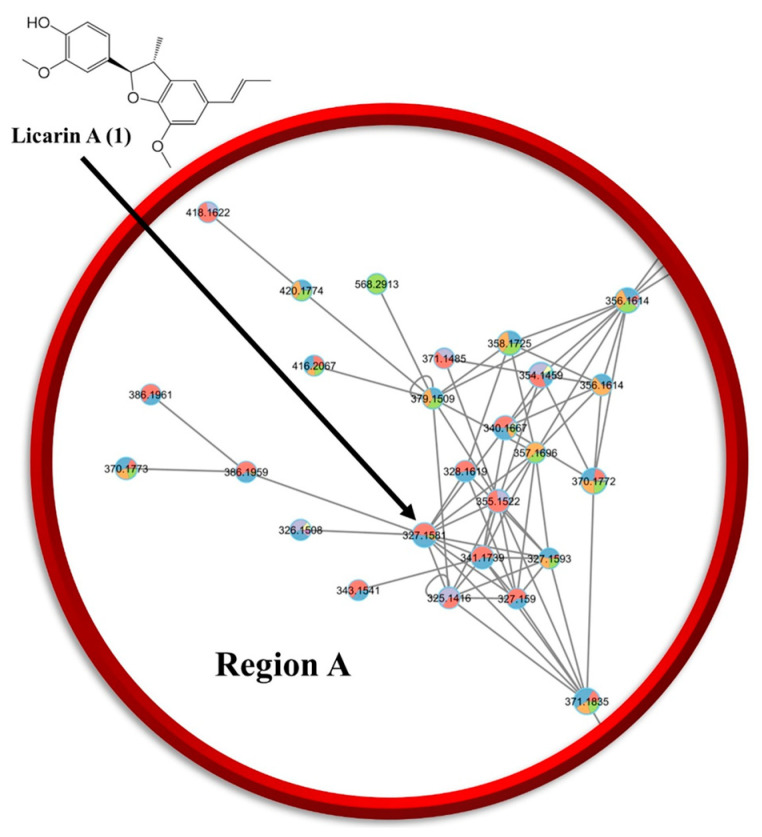
LC-MS/MS chemoinformatic analysis of all chromatographic fractions obtained from the ethyl acetate partition of *M. fragrans* revealed the presence of **1** in region A (see Supplementary Figure S1) of the molecular network. (Relevant data: GNPS library hit, Bronze: 0.81c.)

An ethyl acetate (EA) partition of *Myristica fragrans* was investigated using GNPS in tandem with SIRIUS and analyzed for potential compounds that may be used for cancer chemoprevention (Figure 2 and Supplementary Figure S1). A previous phytochemical report was utilized as a basis to find additional chemical structures present in *M. fragrans* of potential relevance to cancer chemoprevention [7]. As shown in Figure 2, after using ESI-MS/MS in tandem with GNPS and SIRIUS for all extracts and chromatographic fractions of the ethyl acetate partition, the networking analysis conducted pointed towards the prevailing population being lignans [18,19]. Compounds were visualized in a network and compared for similarities. Three regions within the largest cluster as circled on the network that showed a diversified structural set were chosen and depicted as region A, B, or C, from which **1** was identified within region A for further phytochemical studies (Supplementary Figure S1). After acquiring GNPS and SIRIUS data, the top six planar structures that had the highest degree of certainty were chosen from both the GNPS database and the SIRIUS suite of databases to start in silico studies of the compounds.

### 2.2. Bioactivity Screening of M. fragrans Extracts and Fractions

When comparing cell cytotoxicity, phosphoNF-*κ*Bp65 inhibition, and cell oxidative stress of the various *M. fragrans* partitions and fractions, the EA partition and its fraction EAF6 were demonstrated to be the most active (Supplementary Figure S2).

### 2.3. Isolation and Characterization of Licarin A (1)

(+)-Licarin A (**1**) was isolated, characterized, and found to exhibit the same optical rotation, NMR, and MS data as have been reported in the literature previously, most notably from *M. fragrans*, *Nectandra oppositifolia*, and *Machilus odoratissima*, with the (+)-enantiomer having been less studied (Supplementary Figure S3) [14,17,22,23].

### 2.4. Identification of the Binding Site of 1, GNPS Library Hits, and SIRIUS CSI: FingerID Structural Predictions to NF-κBp65 and PARP-1 by Molecular Docking

**Figure 3:**
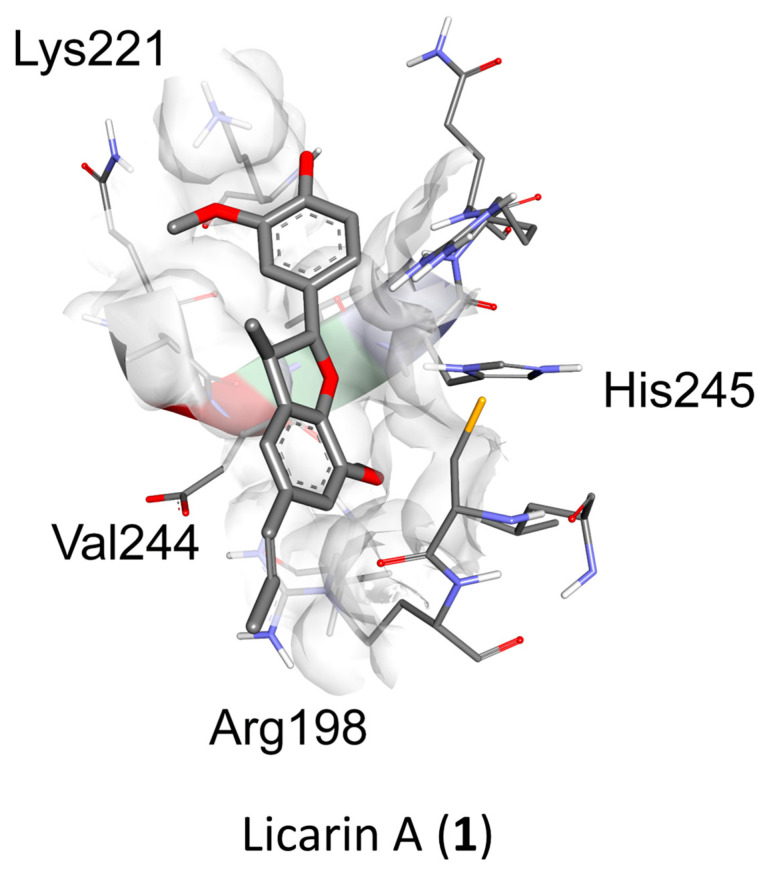
Molecular docking of licarin A (**1**) based on binding energy and structural similarity. The binding pocket of **1** to NF-*κ*Bp65 is shown in a 3D perspective. The 2D perspective displays all binding interactions with relevant amino acids. (Relevant data: in silico binding affinity, 10.66 μM; binding energy, −6.78).

**Figure 4:**
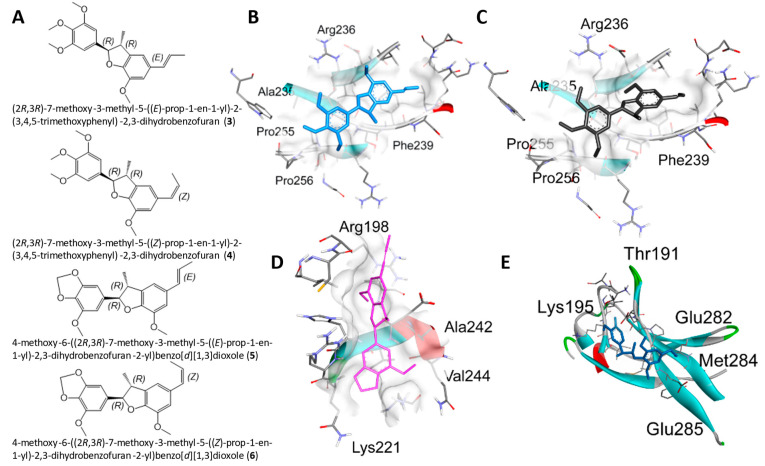
Molecular docking for the SIRIUS CSI: FingerID structural predictions with NF-*κ*Bp65. (**A**) Structures of **3**–**6**. (**B**–**E**) Docking profiles for **3** (blue, **B**), **4** (gray, **C**), **5** (pink, **D**), and **6** (cyan, **E**) with NF-*κ*Bp65.

**Figure 5:**
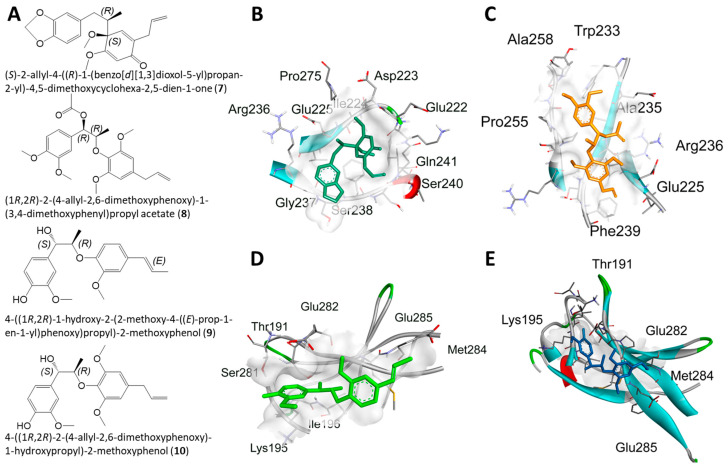
Molecular docking for the GNPS library hits with NF-*κ*Bp65. (**A**) Structures of **7**–**10**. (**B**–**E**) Docking profiles for **7** (dark green, **B**), **8** (yellow, **C**), **9** (neon green, **D**), and **10** (blue, **E**) with NF-*κ*Bp65.

Molecular docking analysis with AutoDockTools 1.5.4 was used to postulate that licarin A (**1**) binds to NF-*κ*Bp65 in a pocket formed by the amino acids Lys-221, His-245, Arg-198, and Val-244, as shown in Figure 3, Figure 4 and Figure 5 and Supplementary Figure S4. The binding affinity (k_i_) of **1** to NF-*κ*Bp65 was calculated as 10.66 μM and was attributed to hydrogen bonding with the amino acids Lys-221 and His-245, π-cation interactions with Arg-198, and π-lone pair interactions with Val-244. The affinity of a structurally similar compounds **3** and **4** correlated to **1** in region A of the molecular network, as shown in Figure 2 and Supplementary Figures S4 and S5, was markedly reduced for the *E*- (k_i_ = 17.9 μM) and the *Z*- (k_i_ = 15.54 μM) stereoisomer. Stereoisomers of two other compounds correlated to **1** in region A of the network—**5** (*E-*, k_i_ = 3.58 μM) and **6** (*Z*-, k_i_ = 7.12 μM)—which displayed improved affinities for NF-*κ*Bp65, as shown in Table 1. Compound **7**, identified in region B of the molecular network, displayed a similarly potent NF-*κ*Bp65 affinity to **5** and **6** with a k_i_ value of 5.3 μM, as shown in Table 1. In contrast, compound **8**, which correlated to **7** in region B, had a reduced affinity to NF-*κ*Bp65 (k_i_ = 37.5 μM). However, compounds **9** and **10** displayed an improved affinity (k_i_ = 8.20 μM and k_i_ = 15.01 μM, respectively) compared to **8**. In comparison, the binding affinity of **1** and the compounds identified in the molecular networking study was much more selective to PARP-1 than NF-*κ*Bp65.

### 2.5. Demonstration of Innocuous Effects by Licarin A in Cell Cytotoxicity and Viability Assays

**Figure 6:**
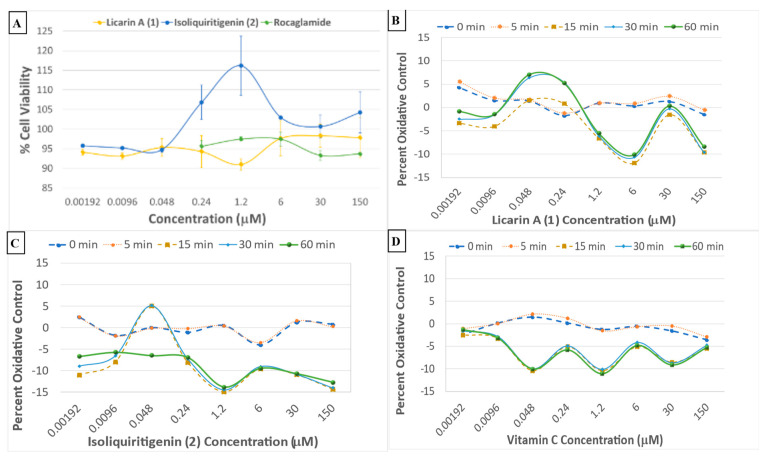
(**A**) Crystal violet viability data for compounds **1** and **2**, and the rocaglamide positive control, with the DU-145 prostate cancer cell line. (**B**–**D**) Cell oxidative stress real-time dose–response assay profiles of **1**, **2**, and vitamin C, respectively, using Hepa1c1c7 mouse hepatoma cells.

When compared to cell viability data (Figure 6A), it could be observed that DU-145 cancer cells remained alive and proliferated, but in a controlled manner by licarin A (**1**), similar to the isoliquiritigenin (**2**) chemopreventive control. In contrast, the positive control rocaglamide maintained the same level of cell viability for the duration of the experiment at concentrations higher than 6 μM.

### 2.6. Comparative Cell Oxidative Control of Licarin A When Compared to 2 and Vitamin C

A cell oxidative stress real-time dose–response assay using Hepa1c1c7 mouse hepatoma cells was used to test all the partitions and fractions from *M. fragrans*. This provided evidence that there was oxidative control by administration of the ethyl acetate partition of chromatographic fractions EAF5–7 (Supplementary Figure S2C). The positive control in the assay, vitamin C, showed a reduction in cellular stress within the first 5 min at 6 μM, showing abrupt degradation of activity thereafter, while both **1** and **2** had a longer-lasting effect, with the activity of **2** (48 nM) degrading after 30 min. In contrast, **1** (48 nM) maintained activity for the entire duration of the assay (Figure 6B–D).

### 2.7. Inflammatory Protection by Licarin A in a phosphoNF-κBp65 Cytosolic Translocation Assay

**Figure 7:**
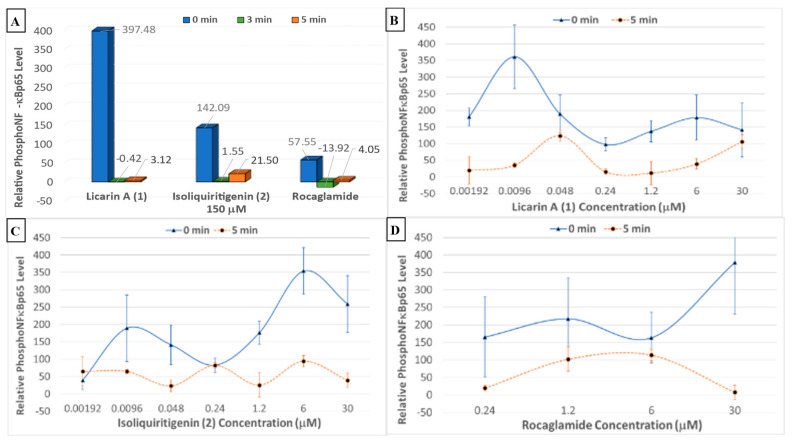
(**A**) Phosphorylated NF-*κ*Bp65 levels after treatment with 150 μM of **1**, **2**, and rocaglamide with the DU-145 prostate cancer cell line. (**B**–**D**) Individual phosphorylated NF-*κ*Bp65 levels after treatment with **1**, **2**, and rocaglamide, respectively, using the DU-145 prostate cancer cell line.

The dried solvent partitions and selected chromatographic fractions from *Myristica fragrans* were also tested for activity in a phosphoNF-*κ*Bp65 cytosolic translocation assay, as shown in Supplementary Figure S2B. The fractions that contained **1** exerted the most potent phosphorylation activity, namely, fractions EAF5–F7. When compared to rocaglamide and the chemopreventive control **2**, licarin A (**1**) afforded superior NF-*κ*Bp65 phosphorylation at a high concentration (150 μM), and enhanced activity at the lower concentration of 9.6 nM in DU-145 prostate cancer cells (Figure 7).

### 2.8. Superior Safety Profile of 1 When Compared to 2 and Tamoxifen

**Figure 8:**
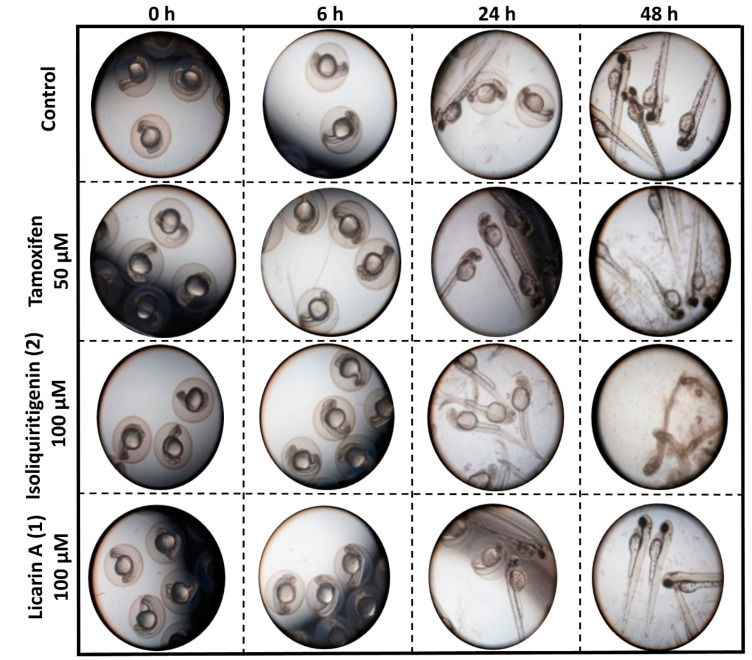
Representative images of the observed morphological toxicity of tamoxifen, licarin A (**1**), and isoliquiritigenin (**2**) in a zebrafish model.

**Figure 9:**
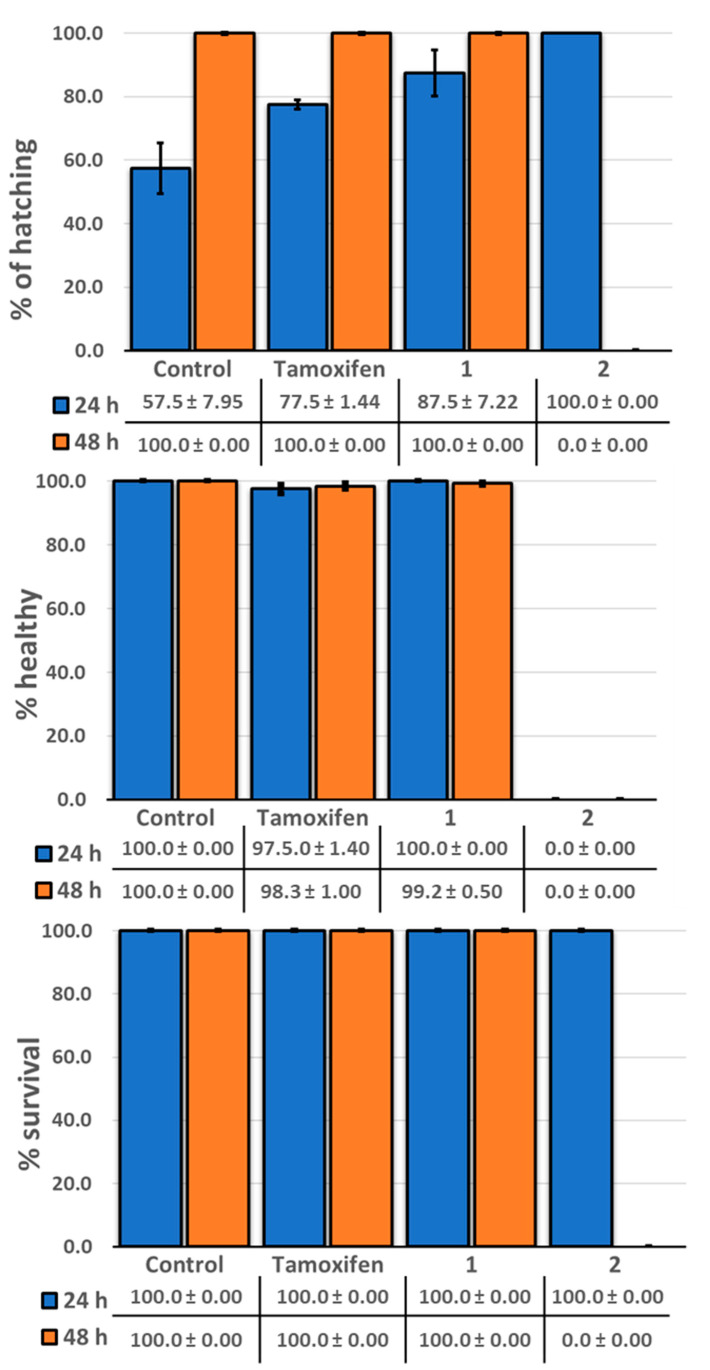
Representative histograms showing the condition of zebrafish after separate exposure to tamoxifen, licarin A (**1**), and isoliquiritigenin (**2**).

The zebrafish model, *Danio rerio*, has been used previously for an initial toxicity screen for bioactive compounds [24]. Therefore, to evaluate the toxicity of licarin A (**1**) in the present study, a cohort of zebrafish larvae was treated separately with a single dose, with isoliquiritigenin (**2**) and the currently approved chemopreventive drug tamoxifen used for comparison [25]. As shown in Figure 8 and Figure 9, when compared to a negative control, the percentages of zebrafish that hatched from the chorions were increased after administration of all three test compounds in the order of decreasing dechorionation percentage value, **2** > **1** > tamoxifen > control.

## 3. Discussion

In a previous report, the arils of *M. fragrans* exhibited chemopreventive activity in vivo using a DMBA-induced mouse skin model when incorporated in the diet [8]. Also, mace aril extracts have shown discernible PARP-1, NF-*κ*B, and lactose dehydrogenase A activities in vitro [7,26]. In the present study, an ethyl acetate partition of *M. fragrans* is being reported for the first time for its innocuous effects at the concentrations tested against the DU-145 cancer cell line for proposed future chemoprevention studies. After further fractionation of this partition, NF-*κ*Bp65 phosphorylation potency was observed to decrease in order of the chromatographic fractions EAF5 > EAF6 > EAF7, which was consistent with the observed abundance of the neolignan constituent licarin A (**1**) in each fraction, as confirmed via LC-MS^2^ and networking analysis (Figure 2 and Supplementary Figure S1). When comparing cell cytotoxicity, NF-*κ*B, and cell oxidative stress of the various *M. fragrans* partitions and fractions, the ethyl acetate partition and its subfraction EAF6 were demonstrated to be the most active (Supplementary Figure S2). This, in turn, correlated well with the networking chemoinformatic analysis conducted that pinpointed **1** in fraction EAF6, with some found also in the surrounding fractions EAF5 and EAF7. Therefore, **1** was isolated in pure form from fraction EAF6 and evaluated for its cell oxidative stress control and NF-*κ*Bp65 phosphorylation activities.

Other structurally similar compounds to licarin A (**1**), identified tentatively in the molecular networking study as GNPS library hits or by SIRIUS CSI: FingerID structural analysis, were analyzed in silico by molecular docking studies to postulate their binding interactions with NF-*κ*Bp65 and PARP-1. The binding pocket of **1** to NF-*κ*Bp65 was determined to encompass hydrogen bonding interactions with the amino acids Lys-221 and His-245, π-cation interactions with Arg-198, and π-lone pair interactions with Val-244, resulting in a calculated binding affinity (k_i_) of 10.66 μM.

The compounds identified from SIRIUS (compounds **3**–**6**) were used for molecular docking (Section 2.4), with the same stereochemistry (*R*) as the isolated licarin A at carbons **2** and **3** in the dihydrofuran ring. The affinity of **1** was markedly reduced for the *E*- (**3**, k_i_ = 17.9 μM) and the *Z*- (**4**, k_i_ = 15.54 μM) stereoisomer in comparison to **1**. As shown in Figure 4 and Supplementary Figure S5, this could be explained by the lack of hydrogen bonding interactions as observed in the binding site for **1**. Instead, the binding pocket of stereoisomers **3** and **4** relies on the π-π stacking interactions with Phe-239, π-lone pair interactions with Arg-236, and π-alkyl interactions the ether functionalities have with Pro-256, Ala-235, and Pro-255. In contrast, stereoisomers **5** (*E-*, k_i_ = 3.58 μM) and **6** (*Z*-, k_i_ = 7.12 μM) displayed an improved affinity for NF-*κ*Bp65. Both stereoisomers bind to NF-*κ*Bp65 in a pocket formed by hydrogen bonding interactions with Lys-221 or Arg-236 to the benzodioxole functionality of **5** or **6**, respectively. In addition, **5** shares π-cation interactions with Arg-198 and π-lone pair interactions with Val-244, while **6** has numerous π-alkyl interactions with Arg-236 and Phe-239. Nevertheless, from these observations it seems that the affinity of the ligand relies on its ability to act as a hydrogen bond acceptor for the positively charged amino acid side chains of the NF-*κ*Bp65 binding site.

The stereochemistry of the GNPS library hits used in the docking study were justified by searching within the LOTUS database for their previous isolation from natural sources. The stereochemistry of the GNPS library hits were verified and chosen if they had previously been isolated using the LOTUS database for subsequent molecular docking studies [27]. For the GNPS library hits **7**–**10**, all have been previously isolated as recorded on the GNPS database. Additionally, the stereocenters for all these compounds are located at carbons 1 and 2. Due to the differing locations to licarin A, we justified the use of the *R* and *S* configuration at carbons **1** and **2**, respectively, for compound **7**, as it has been previously isolated from *M. fragrans* [27]. For compound **8**, the same configuration as licarin A was used for both stereocenters (1*R*, 2*R*), as shown in Figure 5. Compound **8** was chosen with this configuration, as it is located in neighboring region B in the molecular network rather than in region A containing licarin A. Compounds **9** and **10**, also found within region B of the molecular network, possessed the *S* configuration at carbon 1 and the *R* configuration at carbon **2** for the molecular docking simulations in order to assess the activities of the opposing configuration.

The GNPS library hit **7**, identified tentatively in the neighboring region B of the molecular network, displayed a similarly potent affinity to NF-*κ*Bp65 as **1**. Rather than acting as a hydrogen bond acceptor, the benzodioxole functionality, **7** interacted with the positively charged amino acid Arg-236 by π-alkyl interactions, as shown in Figure 5. Additionally, numerous hydrogen bonding interactions formed by the amino acids Ile-224, Gln-241, Asp-223, Pro-275, Glu-222, Ser-240, and Gly-237 most likely contributed to the potent affinity of **7** to NF-*κ*Bp65. The pocket formed between **8** and NF-*κ*Bp65 includes a π-sigma interaction with Ala-235, hydrogen bonding interactions with Arg-236 and Trp-233, and a π-alkyl interaction with Phe-239, as shown in Figure 5. Compound **9** displayed a similar binding affinity to **7** due to a π-sigma interaction with Lys-195; multiple hydrogen bonding interactions with Thr-191, Ser-281, and Ile-196; as well as a π-alkyl interaction with Met-284. The binding affinity of compound **10** was also improved in comparison to **8** likely due to the hydrogen bonding interactions with Arg-246, Gln-247, Lys-221, a π-cation interaction with Arg-246, and π-alkyl interactions with His-245, Leu-215, and Val-244.

In addition, poly(ADP-ribose) polymerase (PARP) inhibitors have been previously suggested as promising chemopreventive options [28]. For instance, PARP inhibitors have been demonstrated to preserve ATP levels, beneficially influence antioxidant status, and normalize the mitochondrial protective protein Bcl-x levels following chemotherapy-induced kidney injury [29,30]. Thus, all compounds identified within the molecular network, including **1**, were investigated by molecular docking to postulate their potential PARP-1 inhibition. As shown in Table 1, the estimated inhibition constant calculated for all of these compounds was in the nanomolar range in the molecular docking to PARP-1, in contrast to the micromolar estimated inhibition activity to NF-*κ*Bp65. This suggests that the potential chemopreventive activity of **1** could be due in part to both the inhibitory activity against the NF-*κ*Bp65 inflammatory pathway and as a PARP inhibitor.

To measure the chemopreventive potential of **1**, two major processes that are implicated in cancer incidence were evaluated, involving reactive oxygen species generation and inflammation [7]. ROS can cause mitochondrial and DNA damage, leading to mitochondrial malfunction and, finally, cancer cell viability [31]. Therefore, compounds that are able to eliminate ROS may play an important role in chemoprevention by delaying the onset of some cancer types and their development [20,32]. In addition, the inflammatory transcription factor, NF-*κ*B, has a role in cell proliferation and survival, as well as regulation of the cell cycle, and is a known sensor of oxidative stress by detecting ROS at low levels [30]. An increase in ROS has been implicated in the development of cancer through the regulation of NF-*κ*B activating pathways [31]. Additionally, NF-*κ*B experiences an autofeedback loop through the I*κ*Bα, which is newly synthesized and causes an oscillating activity curve [33]. Therefore, **1** was tested for cell oxidative stress and phosphorylation of NF-*κ*Bp65.

Previously, **1** was demonstrated to have potent antioxidant activity in the DPPH radical scavenging assay [15]. Therefore, to study further how **1** relates to cancer chemoprevention, an oxidative control assay was used to compare this neolignan with a natural cancer chemoprevention control product, compound, **2**, which has been demonstrated to induce mitochondrial deterioration and apoptosis in a ROS-dependent manner [4,20,34]. Vitamin C is regarded as an antioxidant “gold standard” and, therefore, was employed as a positive control in this assay [31]. When tested, vitamin C exerted potent oxidative control, although it lost its protective effects after 5 min (Figure 6D). When compared to vitamin C, **2** had a longer-lasting effect, but the activity degraded after 30 min while **1** maintained activity for the entire duration of the assay (60 min) at 48 nM (Figure 6B–D). An in silico evaluation against the chemoprevention marker NF-*κ*Bp65 indicated **1** to have an affinity towards the NF-*κ*Bp65 binding pocket located in the middle of chains A and B (Figure 3 and Supplementary Figure S4). This would increase the binding strength to the protein inducing NF-*κ*B phosphorylation while minimally affecting cell viability, as phosphoNF-*κ*Bp65 is being prepared for ubiquitination [33]. Therefore, the results obtained for **1** afforded a large window between the cytotoxic concentration in vitro (IC_50_ = 100.06 μM: Supplementary Figure S7) and that of in silico molecular docking (k_i_ = 10.66 μM; Δ*G* = –6.78; Table 1). This shows promise due to the more potent effective concentrations of **1** of 9.6 nM and 48 nM determined for inducing NF-*κ*B phosphorylation or oxidative control than for either **2** or the rocaglamide and vitamin C controls, respectively, over an extended period. Since rocaglamide has been demonstrated to be an inducer of NF-*κ*B protein levels in the cytoplasm while inhibiting NF-*κ*B phosphorylation levels in the nucleus and cytoplasm, it was used as the positive control in this assay [35]. Licarin A (**1**), therefore, demonstrated oxidative control and NF-*κ*B pathway activation at low nanomolar concentrations, and had an improved profile compared to the positive controls used long term.

As shown in Figure 6A,B and Figure 7A, at a concentration of 150 μM, higher than the cytotoxic dose (IC_50_ = 100.06 μM), **1** increased both the phosphorylation of NF-*κ*Bp65 and the production of reactive oxygen species. At time point 0 min, this correlates with inflammatory protection via phosphoNF-*κ*Bp65 as it is prepared for ubiquitination and a ROS response associated with cell damage while maintaining cell viability confirmed after 5 min, at which point the phosphoNF-*κ*Bp65 levels decreased [33]. However, when lower than the cytotoxic concentration, **1** had a comparable effect on both targets. For instance, at concentrations lower than that of the cytotoxic dose (48, 9.6, and 1.92 nM), there was an immediate acute increase in phosphorylated NF-*κ*Bp65 that was sustained at 48 nM (Figure 7D). This contrasts with **2**, which did not sustain its initial response at any of these lower doses for the duration of the experiment (Figure 6C). Furthermore, at a nearly 100× lower concentration of **1** (9.6 nM), a higher-percent phosphorylation of NF-*κ*Bp65 was induced compared to **2** at 6 μM. Also, at a dose of 1.2 μM, cell viability was slightly diminished at the same dose, which corresponds to an increase in ROS production and minimal NF-*κ*Bp65 phosphorylation at 0 min compared to that of 9.6 nM. Conversely, as the protective effects of **1** increased, cell viability was maintained, namely, at 9.6 nM and 48 nM. Further, in comparison to rocaglamide, **1** had a similar duration of effect at concentrations above 0.24 μM, with the exception of 30 μM, at which **1** did induce phosphorylation of NF-*κ*Bp65 despite the increase in reactive oxygen-level species production at the same doses (Figure 7B,D). The phosphorylation of NF-*κ*Bp65 and long-term oxidative control capabilities suggest that **1** exerts its effects by acting on both molecular processes, and hence it may be conjectured that the extended use of **1** could lead to prolonged protective effects possible at a lower dose.

As shown in Figure 8 and Figure 9, a 50 μM concentration of the clinically utilized drug tamoxifen as positive control was well tolerated in the zebrafish (*Danio rerio*) model used, until 48 h after administration, at which point these organisms displayed certain developmental abnormalities, including a curved or shortened spine, with this drug exhibiting the second highest number of abnormalities of the three compounds tested, consistent with similar results to a previous report [36]. In contrast, the neolignan **1** was well tolerated by the zebrafish at a higher dose of 100 μM after 48 h, as evidenced by both their 100% survival and lack of morphological changes. On the other hand, administration of 100 μM of **2**, a natural cancer chemopreventive control product, was toxic to the zebrafish as early as 24 h after administration, with all fish in the cohort displaying curved spines or shortened tails with 100% hatching, resulting in their death before 48 h, similar to previously published results [21]. This demonstrates the potential improved safety profile of **1** relative to **2**.

Finally, it should be noted that several other neolignans have been documented previously as cancer chemopreventive agents, including ailanthoidol [37] honokiol [38], and magnoliol [39]. In addition, in contrast to licarin A (**1**), which did not produce any discernible growth inhibitory activity against DU-145 human prostate cancer cells in the present study, its trimethoxylated analog licarin C, when obtained from *M. fragrans*, was shown to be potently cytotoxic against HT-29 human colon cancer cells in the nanomolar range [7].

## 4. Materials and Methods

### 4.1. General

^1^H, ^13^C, DEPT, HSQC, and HMBC NMR spectra were measured using a Bruker Avance 400 MHz spectrometer. The NMR spectra were recorded in chloroform-*d* (Cambridge Isotope Laboratories, Tewksbury, MA, USA), with tetramethylsilane used as an internal standard. Molecular weights were obtained using a Waters Q-ToF micro mass spectrometer and a Bruker maXis Q-ToF mass spectrometer. The specific rotation of licarin A (**1**) was recorded at 25 °C on an Anton Paar MCP 15 polarimeter (Anton-Paar, Ashland, VA, USA), using MeOH at the sodium (D) wavelength (589 nm).

Open column chromatography was performed, using a silica gel 60 F_254_ (70–230 mesh) (GE Healthcare, Piscataway, NJ, USA). Methanol and acetonitrile (Fisher Scientific, Fair Lawn, NJ, USA) were used for extraction and isolation. Thin-layer chromatography (TLC) F_254_ plates (Merck, Darmstadt, Germany) were utilized to monitor separations. The TLC plates were developed using a solution of 10% H_2_SO_4_ in ethanol containing 1% vanillin and were heated to visualize the separations.

### 4.2. Plant Material

The dried arils of *Myristica fragrans* Houtt. (Myristicaceae) (mace) were obtained from Sabinsa Corporation, East Windsor, NJ, USA (batch number RD/Mace/02). A voucher specimen (number: mace 1500) has been deposited at the College of Pharmacy, Ohio State University.

### 4.3. Extraction, Isolation, and Compound Identification

The extraction of *M. fragrans* arils (1.2 kg) was performed with ethyl acetate (EA), as published previously [7]. The impure fraction EAF6 containing licarin A (**1**) (50 mg) was purified by silica gel column chromatography (230–400 mesh), with a solvent gradient of hexane-CH_2_Cl_2_ (100:0 to 5:95, *v*/*v*), to afford 58 subfractions (EAF6F1-F58), which were pooled according to TLC analysis. From combined fractions EAF6F8-F14, **1** was obtained as a pure white solid (30 mg). The purity of this isolated compound was confirmed by TLC profiling under UV light and by visualization with H_2_SO_4_-vanillin solution, and its identity was confirmed by ^1^H and ^13^C NMR spectroscopy and HRESIMS, in addition to measurement of its optical rotation (Supplementary Methods Section S1.1).

### 4.4. Test Compounds and Reagents

Paclitaxel (CAS: 33069-62-4) was obtained from MedKoo Biosciences, Inc. (Durham, NC, USA). Rocaglamide (CAS: 84573-16-0) was obtained from Sigma-Aldrich (St. Louis, MO, USA). Tamoxifen (CAS: 10540-29-1) was purchased from Sigma-Millipore (St. Louis, MO, USA). Isoliquiritigenin (**2**) was isolated from *Glycyrrhiza glabra* (licorice) and synthesized in pure form previously in our laboratory [20].

### 4.5. Cell Culture

The DU-145 *Homo sapiens* prostate cancer and Hepa1c1c7 *Mus musculus* hepatoma cell lines were obtained from the American Type Culture Collection (ATCC, Manassas, VA, USA). Cells were cultivated as monolayers in T75 tissue culture flasks and kept in a humidified incubator at 37 °C with 5% CO_2_. Dulbecco Modified Eagle’s Medium (DMEM) or Modified Eagle’s Medium (MEM) [(−) nucleoside] were used and supplemented with 10% fetal bovine serum (FBS) and a 1% antibiotic-antimycotic mixture containing penicillin and streptomycin (Gibco, Rockville, MD, USA) for the human and mouse cancer cells, respectively. Test compounds were dissolved in dimethyl sulfoxide (DMSO) (CAS 67-68-5) from Sigma-Aldrich, and diluted compound concentrations were prepared in ddH_2_O:DMSO (9:1). Furthermore, the control group was treated with ddH_2_O: DMSO (9:1). Thus, the final concentration of DMSO was 0.2% for cell-based assays and 0.03% for immunohistochemistry and zebrafish in vivo assays.

### 4.6. Feature-Based Molecular Networking (FBMN), Global Natural Product Social Molecular Networking (GNPS), and SIRIUS Analysis

The LC-MS^2^ data acquisition, parameters, and MZmine 3 data preprocessing information is provided in the Supplementary Methods Sections S1.2 and S1.3 [40]. Mass spectrometric data were processed using MZmine 3, and the resulting values were exported to GNPS for FBMN analysis [18,41]. To filter the data, ions from solvent blank samples and all MS/MS fragment ions within ±17 Da of the precursor *m/z* value were removed. MS/MS spectra were window-filtered by selecting only the top six fragment ions in the ±50 Da window through the spectrum. The precursor ion mass tolerance was set to 2 Da, and the MS/MS fragment ion tolerance was set to 0.02 Da.

A network was created, and the edges were filtered based on their cosine score, keeping only those with a score of greater than 0.7 and more than six matched peaks. The edges between two nodes were retained only if each node was in the other top 20 most similar nodes. The maximum molecular family size was limited to 120, and edges with the lowest scores were removed until the size was below this threshold. The MN was then visualized using Cytoscape 3.9.1 (Figure 2 and Supplementary Figure S1) [42].

The MS/MS data (.mgf file) were processed with the bioinformatic tool SIRIUS 5.8.6, including CSI:FingerID and CANOPUS for chemical class and compound annotations, molecular formula prediction with ZODIAC, and COSMIC to assign confidence to structure annotations [19,43,44,45,46,47]. The MN was then visualized using Cytoscape 3.9.1 (Figure 2 and Supplementary Figure S1) [42].

The MS/MS spectra were searched against GNPS spectral libraries, with matches required to have a score above 0.7 and at least six matched peaks [18,48]. Additional edges were included as well. The MN job can be accessed at http://gnps.ucsd.edu/ProteoSAFe/status.jsp?task=f83f6b3198ba46b69f227b01a0e9b733 (accessed on 12 March 2023).

### 4.7. Molecular Docking

Molecular docking of compounds **1** and **3**–**10** with NF-*κ*Bp65 and PARP-1 was performed using crystallographic structures (PDB: 1MY5 and 5DS3, respectively) obtained from the Protein Data Bank. The stereochemistry of the SIRIUS and GNPS library hits used for molecular docking simulations are expanded on further in the discussion. The structure of the protein was prepared by adding all hydrogen (polar and no polar) and Kollman charges using AutoDock Tools 1.5.4 [48,49,50,51]. The starting conformation of the ligands was an energy-minimized form obtained after a geometric optimization using a semi-empirical force field, as implemented in Spartan 10. Compound **1** was evaluated by assigning the Gasteiger–Marsilli atomic charges and non-polar hydrogens obtained with AutoDockTools 1.5.4 [49].

Docking: Blind docking was carried out with AutoDock4 software (http://autodock.scripps.edu/ accessed on 12 March 2023) using the default parameters, except for the number of GA runs (100) and the Lamarkian genetic algorithm with a local search and 25 million energy evaluations (Long. Evals.) per run. The protein was held rigid during the docking process while ligands were allowed to be flexible. The grid box size was 126 × 126 × 126 points (0.375 Å each) in the x, y, and z dimensions, with the center of the grid corresponding to the protein. The predicted docked complexes (protein–ligand) were the conformations showing the lowest binding energy. Estimated inhibition constants (k_i_) were calculated from the docking energy displayed by AutoDock following the equation k_i_ = $e^{\frac{\Delta G\times 1000}{RT}}$, where Δ*G* is the docking energy, R is the universal constant of an ideal gas (1.98719 cal K^–1^ mol ^–1^), and T is the temperature (298.15 K).

### 4.8. SRB Cell Cytotoxicity Assay

The antiproliferative effects of **1**, the chemopreventive control **2**, and the positive control paclitaxel were measured using a sulforhodamine B (SRB) cytotoxicity assay [52]. Briefly, DU-145 prostate cancer cells (5 × 10^4^ cells/well) were seeded in a 96-well plate overnight. Then, the cells were treated at different concentrations ranging from 0 to 150 μM with each test compound for 24, 48, and 72 h (Supplementary Figure S7). After being treated, the cells were incubated with cold trichloroacetic acid (20%, 30 min), washed with tap water, stained with SRB (0.4%, 30 min), and washed subsequently with acetic acid (1%). Finally, the unbound dye was solubilized using 200 μL of Tris-base (10 mM) and the absorbance read at 515 nm using a FLUOstar Optima microplate reader (BMG Labtech Inc., Durham, NC, USA). The percent inhibition was determined by using the formula provided in the prior publication.

### 4.9. Cell Viability Crystal Violet Assay

The cell viability of DU-145 prostate cancer cells was evaluated after treatment with **1**, **2**, and the positive control rocaglamide using the protocol from a Phospho-NF-kappaBp65 (S468 + S536) and Total In-Cell ELISA Kit (ab207482) (Abcam Inc., Waltham, MA, USA), after initial PhosphoNF-*κ*Bp65 translocation steps (Section 4.11).

### 4.10. Cell Oxidative Stress Assay

A reactive oxygen species (ROS) assay was performed following a previously described procedure [53]. The generated intracellular levels of ROS were measured using the fluorescent probe 2′,7′-dichlorodihydrofluorescein diacetate (DCFH-DA). Hepa1c1c7 mouse hepatoma cells were seeded in a 96-well plate, treated with **1**, **2**, or the positive control rocaglamide, followed by 5 h incubation at 37 °C with 5% CO_2_. Subsequently, cells were incubated with H_2_O_2_ (1.25 mM) and FeSO_4_ for 30 min at 37 °C. Later, the fluorescent probe DCFH-DA was added to determine intracellular ROS. Fluorescence was measured using a FLUOstar Optima fluorescence plate reader (BMG Labtech Inc., Durham, NC, USA) at an excitation wavelength of 485 nm and emission wavelength of 530 nm. All treatments performed in triplicate were representative of at least two different experiments.

### 4.11. PhosphoNF-κBp65 Assay

PhosphoNF-*κ*BP65 phosphorylation levels were evaluated using the protocol from an Abcam Phospho-NF-kappaBp65 (S468 + S536) + Total In-Cell ELISA Kit (ab207482) (Abcam Inc., Waltham, MA, USA). The effects of **1**, the natural product chemopreventive positive control **2**, and a rocaglamide positive control in the PhosphoNF-*κ*Bp65 assay activity were measured using a FLUOstar Optima plate reader. DU-145 or Hepa1c1c7 cells (5 × 10^4^ cells/well) were seeded in a white 96-well plate and treated (0–150 μM) for 5 h. Afterwards, luminescence was measured using a FLUOstar Optima plate reader.

### 4.12. Toxicity Assay in Zebrafish

Toxicity evaluations on **1**, along with the natural product chemopreventive control **2** and the clinically used chemopreventive agent tamoxifen, were performed using zebrafish (*Danio rerio*) as an animal model by a trained laboratory investigator [24,54,55,56]. Animals were provided by the Department of Neuroscience at The Ohio State University (OSU) (OSU IACUC-approved animal protocol number 2014A00000006-R1-AR1). Zebrafish embryos were housed in aquaculture water at 28 °C with a 14 h/10 h light/dark cycle. After 24 h post fertilization (hpf), groups of random embryos were exposed to different concentrations (0, 50, or 100 μM, *n* = 25) for 24 and 48 h. Then, images of the zebrafish were taken using an Axiovert 40 CFL Zeiss microscope and ProgRes C10 plus camera. Morphological changes in zebrafish development were determined and recorded.

### 4.13. Statistical Analysis

All data obtained represent the means ± standard error of the mean (SEM) of triplicates.

## 5. Conclusions

The present study shows that the neolignan licarin A (**1**) is worthy of further evaluation for its chemopreventive potential, especially because of its lack of toxicity. In particular, it was observed that **1** was highly tolerable in vivo to zebrafish with a lack of morphological changes at a high dose. The observations described in this paper of the higher treatment concentration required for the observed effects for **1** could be due to the somewhat insoluble nature of this neolignan, as found for certain other natural products [57]. In a follow-up investigation, it is intended that **1** will be evaluated in a new cancer chemopreventive model designed using zebrafish in order to test the capability of this neolignan in preventing any tumorigenic occurrence in response to environmental carcinogenic exposure in vivo, involving a reversal of mutagen-induced protein level changes. In addition, the influence of **1** on the NF-*κ*Bp65 and PARP signaling cascade will be explored further.

## Figures and Tables

**Table 1:** The collected molecular docking data for NF-*κ*Bp65 and PARP-1 as well as the molecular networking identification method (GNPS or SIRIUS) and calculated certainty coefficients for licarin A (**1**) and compounds **3**–**10**.

Compound No.	Estimated Free Energy of Binding (kcal/mol)	Estimated Inhibition Constant, k_i_ (µM)	GNPS vs. SIRIUS Database (Tier)	Certainty Coefficient
NF-*κ*Bp65				
Licarin A (1)	−6.78	10.66	GNPS (Bronze)	0.81 c
3	−6.48	17.9	SIRIUS	80.60%
4	−6.56	15.54	SIRIUS	80.60%
5	−7.43	3.58	SIRIUS	90.70%
6	−7.02	7.12	SIRIUS	90.70%
7	−7.20	5.30	GNPS (Bronze)	0.82 c
8	−6.04	37.5	GNPS (Bronze)	0.90 c
9	−6.93	8.20	GNPS (Bronze)	0.87 c
10	−6.58	15.01	GNPS (Bronze)	0.82 c
PARP-1				
Licarin A (1)	−8.93	0.286	GNPS (Bronze)	0.81 c
3	−8.45	0.639	SIRIUS	80.60%
4	−8.49	0.598	SIRIUS	80.60%
5	−8.79	0.363	SIRIUS	90.70%
6	−8.86	0.363	SIRIUS	90.70%
7	−9.5	0.108	GNPS (Bronze)	0.82 c
8	−8.56	0.530	GNPS (Bronze)	0.90 c
9	−8.90	0.300	GNPS (Bronze)	0.87 c
10	−9.43	0.123	GNPS (Bronze)	0.82 c

For all the compounds, including **1**, the estimated inhibition constant was in the nanomolar range for PARP-1, with the exception of **10,** in contrast to the micromolar range of activity for NF-*κ*Bp65, as shown in Table 1.

## Data Availability

Data related to results reported in this article can be obtained from the authors upon reasonable request.

## Supplementary Materials

The following supporting information can be downloaded at: https://www.mdpi.com/article/10.3390/molecules29204919/s1, Figure S1: LC-MS/MS chemoinformatic analysis of all chromatographic fractions obtained from the ethyl acetate partition of *M. fragrans*; Figure S2: *M. fragrans* solvent partitions and chromatographic fractions when evaluated against two cancer cell lines; Figure S3: ^1^H and ^13^C NMR spectra of licarin A (**1**) after isolation and purification; Figure S4: Binding interactions of both stereoisomers of compound **1** with relevant amino acids of NF-κBp65; Figure S5. Binding interactions of compounds **3**, **4**, **5**, and **6** identified in region A of the molecular network with relevant amino acids of NF-κBp65; Figure S6. Binding interactions of compounds **7**, **8**, **9**, and **10** identified in region B of the molecular network with relevant amino acids of NF-κBp65; Figure S7. Comparative cancer cell cytotoxicity evaluation of licarin A (**1**) and paclitaxel against the DU-145 prostate cancer cell line.
