# Supplementary material for: Molecular Networking, Docking, and Biological Evaluation of Licarin A from Myristica fragrans as a Potential Cancer Chemopreventive Agent
Source: Molecules. 2024 Oct 17;29(20):4919. doi: 10.3390/molecules29204919 (PMC11510329; doi:10.3390/molecules29204919)
Supplement: Supplementary file 1 [file molecules-29-04919-s001.zip › molecules-3210059-supplementary.pdf]

SUPPLEMENTARY METHODS SECTION

1.1. Physical and spectroscopic data obtained for purified licarin A (1)

The pure compound licarin A (**1**) was obtained as a white solid; $[\alpha]_D^{20}$+27 (*c* 1.0, MeOH); ^{1}H NMR (400 MHz, $CDCl_3$) δ 6.95 (1H, d, *J* = 1.3 Hz, H-2), 6.88 (2H, s, H-6), 6.88 (s, 2H, H-5), 6.77 (s, 1H, H-2′), 6.75 (s, 1H, H-6′), 6.34 (1H, dd, *J* = 1.6, 15.7 Hz, H-7′), 6.09 (1H, qd, *J* = 6.6, 15.7 Hz, H-8′), 5.08 (1H, d, *J* = 9.4 Hz, H-7), 1.85 (3H, dd, *J* = 1.6, 6.6 Hz, H-9′), 1.36 (3H, d, *J* = 6.8 Hz, H-9). ^{13}C NMR (400 MHz, $CDCl_3$) 146.7 (C-3), 146.6 (C-4′), 145.8 (C-4), 144.2 (C-3′), 133.2 (C-5′), 132.2 (C-1), 132.0 (C-1′) 130.9 (C-7′), 123.5 (C-8′), 120.0 (C-6), 114.1 (C-5), 113.3 (C-6′), 109.3 (C-2′), 108.9 (C-2), 93.8 (C-7), 56.1 (OCH_3), 56.0 (OCH_3), 45.6 (C-8), 18.4 (C-9′), 17.6 (C-9) (Supplementary Figure 3). HRESIMS *m/z* 327.1581 $[M + H]^+$ (calcd for $C_{20}H_{23}O_4$ 327.1596). TLC R_f = 0.33, $CHCl_3$-hexanes (3:7).

1.2. LC-MS^2 analysis

Chromatographic fractions obtained from the ethyl acetate partition of *M. fragrans* were analyzed using an UltiMate 3000 UHPLC system, coupled to a Q-Exactive plus hybrid quadrupole Orbitrap spectrometer (Thermo Fisher Scientific, USA). An ACQUITY UPLC BEH C_{18} column (130 Å 2.1 × 100 mm, 1.7 µm, Waters, MA, USA) was used to analyze the samples with an eluent mixture containing water -0.1% formic acid (A) and acetonitrile -0.1% formic acid (B) as the mobile phase. A gradient elution program was used as follows: 0 min, 80% A and 20% B; 13.5 min to 15 min, 2% A and 98% B; 16 min to 18 min, 80% A and 20% B. The flow rate was 400 µL/min. The column compartment temperature was 40 °C and the injection volume was 5 µL.

The MS parameters were set as follows: positive-ion mode; spray voltage: - 3.5 kV; sheath gas flow rate: 50 arbitrary units (arb); auxiliary gas: 13 arb; capillary temperature: 263 °C; S-lens RF level: 50 V; scan mode: full scan (resolution 70,000 FWHM) and dd-MS^2 (resolution 17,500 FWHM); scan range: *m/z* 100-1500. The normalized collision energy (NCE) was set at 30%. The top five most abundant ions in each scan cycle were selected as precursor ions to obtain their MS/MS spectra. Data were processed using Xcalibur™ 4.1 software (Thermo Fisher).

1.3. Data preprocessing with MZmine 3 software

The MS^2 data files from three samples were transformed into .mzML format using MSConvert software. MZmine 3 was then used to process all .mzML files. The mass detection noise level was set to 1.0E5 and 10 for MS level 1 and MS level 2, respectively. The ADAP chromatogram builder was employed with a minimum group size of three scans, a group intensity threshold of 1.0E3, a minimum highest intensity of 1.0E5, and an *m/z* tolerance of 0.0015 (or 5 ppm). An ADAP featured resolver module was used with S/N threshold = 6, minimum feature height = 1.0E5, coefficient/area threshold = 100, peak duration range 0.05 - 1.00 min, and RT wavelet range 0.01-0.05 min. MS^2 scans were paired with an *m/z* tolerance range of 0.0015 (or 5 ppm) and a RT tolerance of 0.15 min. Isotopologues were grouped using the isotopic peak algorithm with an *m/z* tolerance of 0.0015 (or 5 ppm) and an RT tolerance of 0.03 min. A join aligner module was utilized for peak alignment with *m/z* tolerance = 0.0015 (or 5 ppm), weight for *m/z* = 1, RT tolerance 0.1 min, weight for RT = 1. A raw filter was applied to retain features that were present in at least one of the three analyzed samples. The peak list was gap-filled using the peak finder module with a peak shape tolerance of 10%, a *m/z* tolerance = 0.0015 (or 5 ppm), and 0.1 min. The

metaCorrelate module was then used (RT tolerance = 0.015, intensity correlation threshold = 1.0E5, and default correlation grouping parameters), followed by the ion identity module for MS positive mode with an *m/z* tolerance = 0.0015 (or 5 ppm) and a maximum cluster size of 3. The GNPS Export module was employed to export the MS^2 spectral data file (.mgf), the peak quantification table file (.csv), and the annotation edge file (.csv) for the most intense spectra.

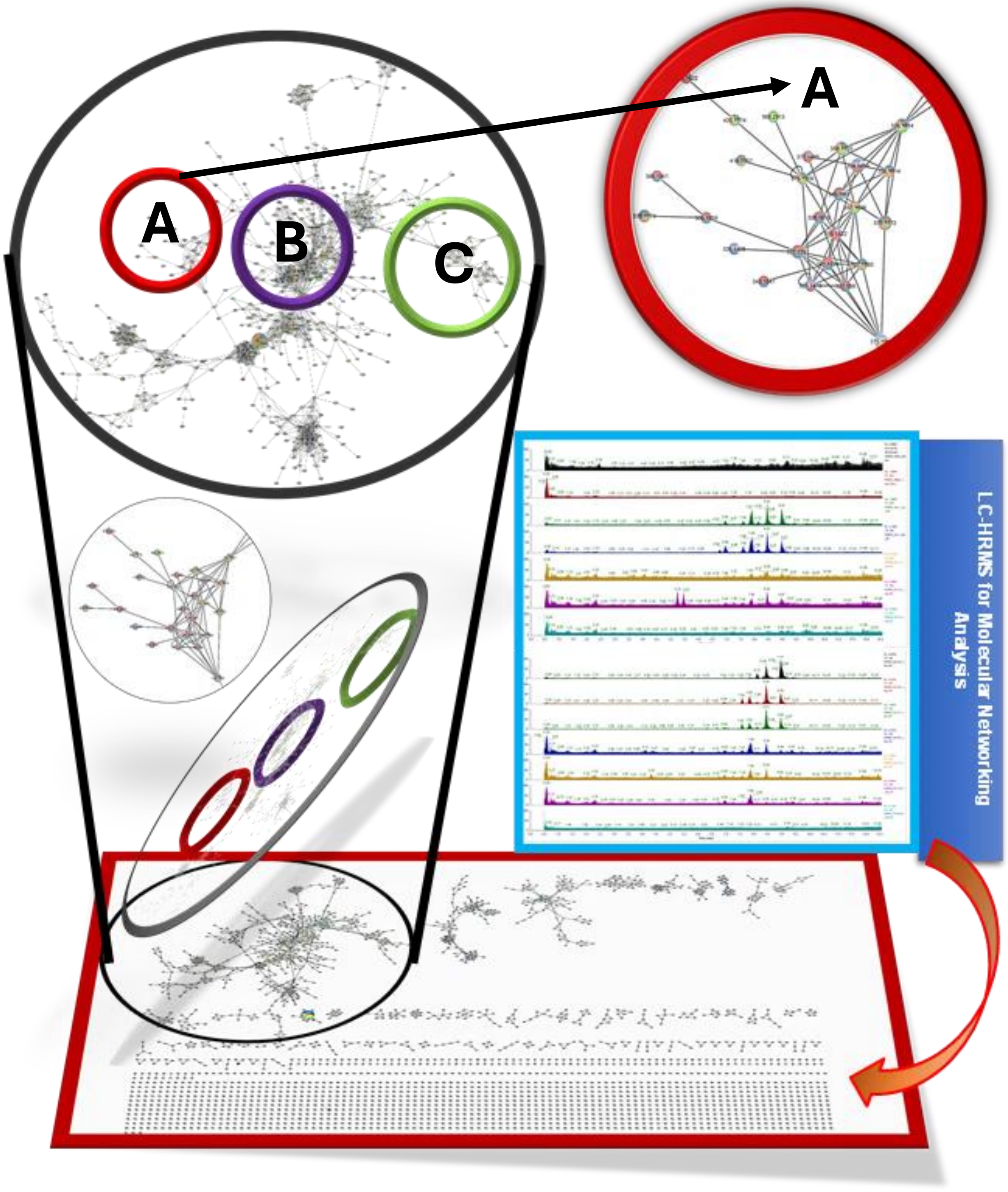

Supplementary Figure S1. LC-MS/MS chemoinformatic analysis of all chromatographic fractions obtained from the ethyl acetate partition of *M. fragrans*.

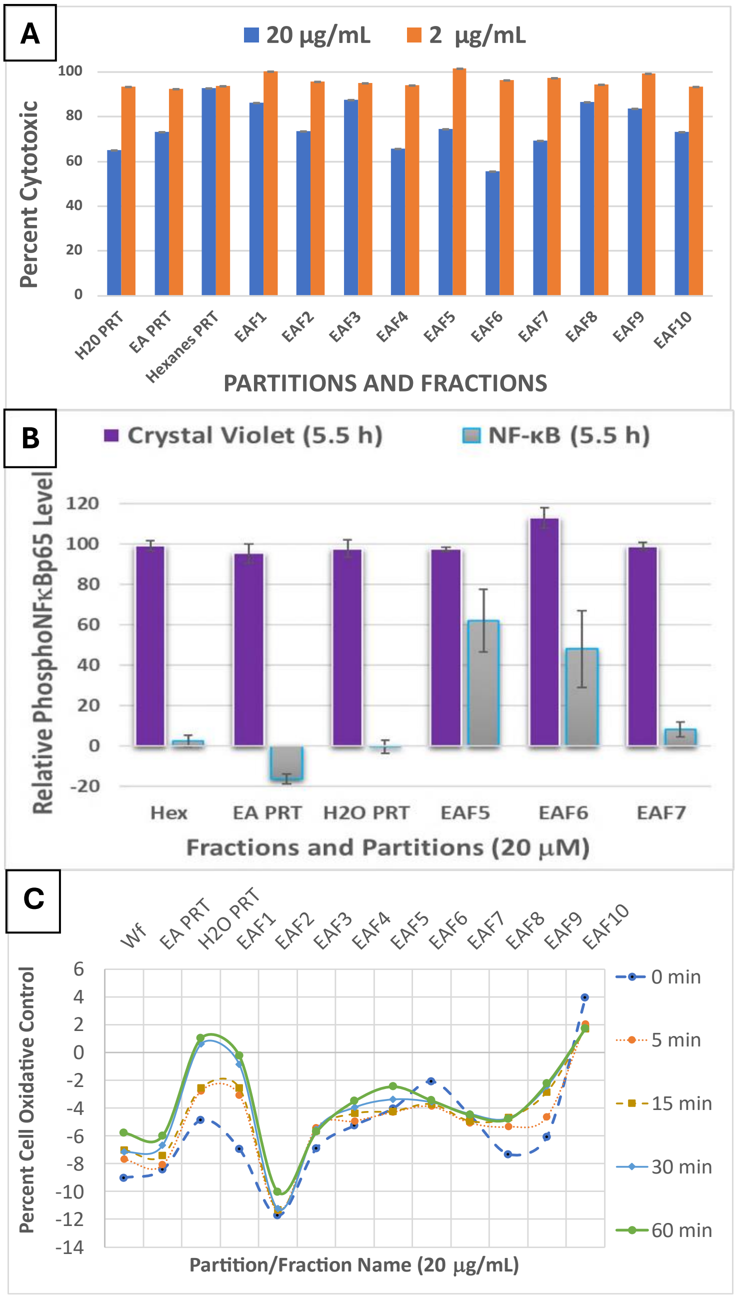

Supplementary Figure S2. *M. fragrans* solvent partitions and chromatographic fractions when evaluated against two cancer cell lines. (**A**) SRB cytotoxicity assay using DU-145 prostate cancer cells. (**B**) Crystal violet cell viability assay and phosphorylated NF-κBp65 levels after treatment using DU-145 prostate cancer cells. (**C**) *M. fragrans* partitions and fractions in a cell oxidative control assay using the Hepa 1c1c7 mouse hepatoma cell line.

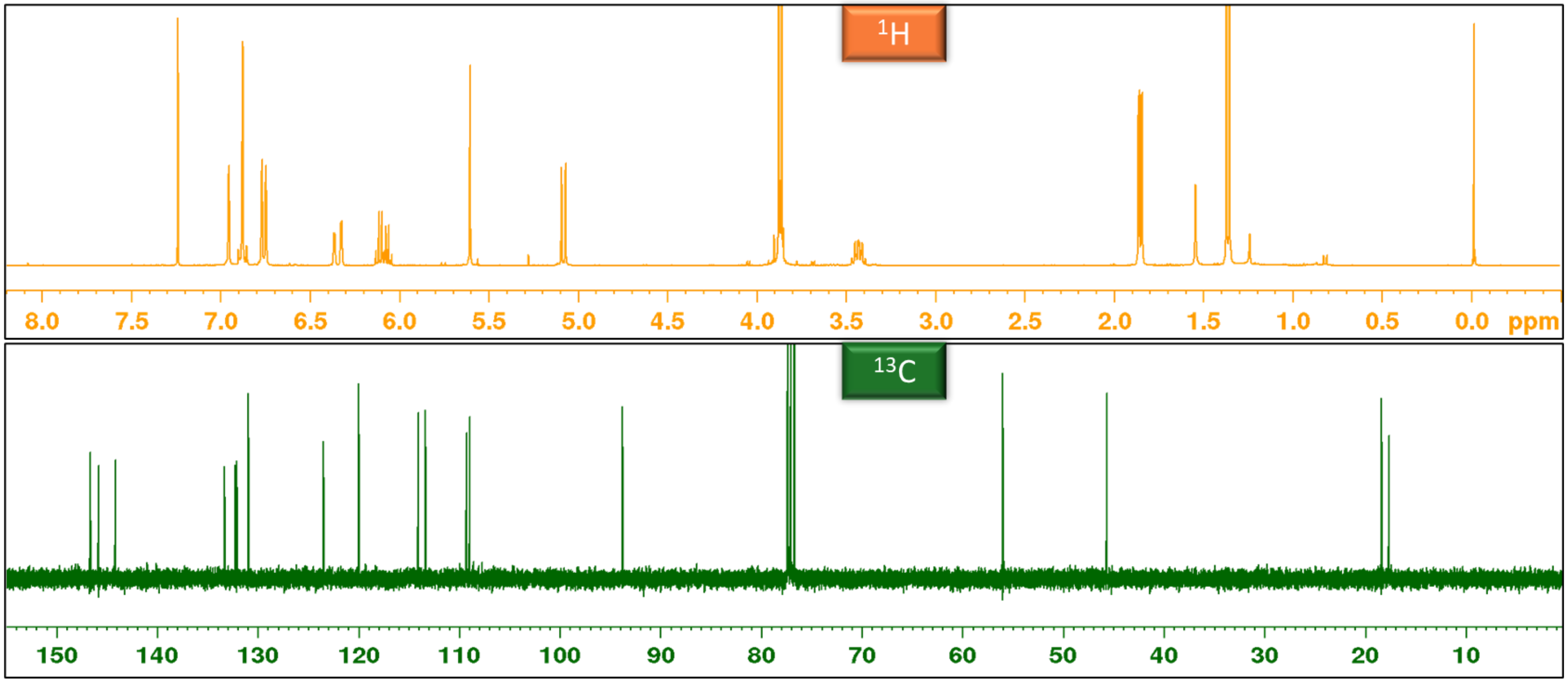

Supplementary Figure S3. ^{1}H and ^{13}C NMR spectra of licarin A (**1**) after isolation and purification.

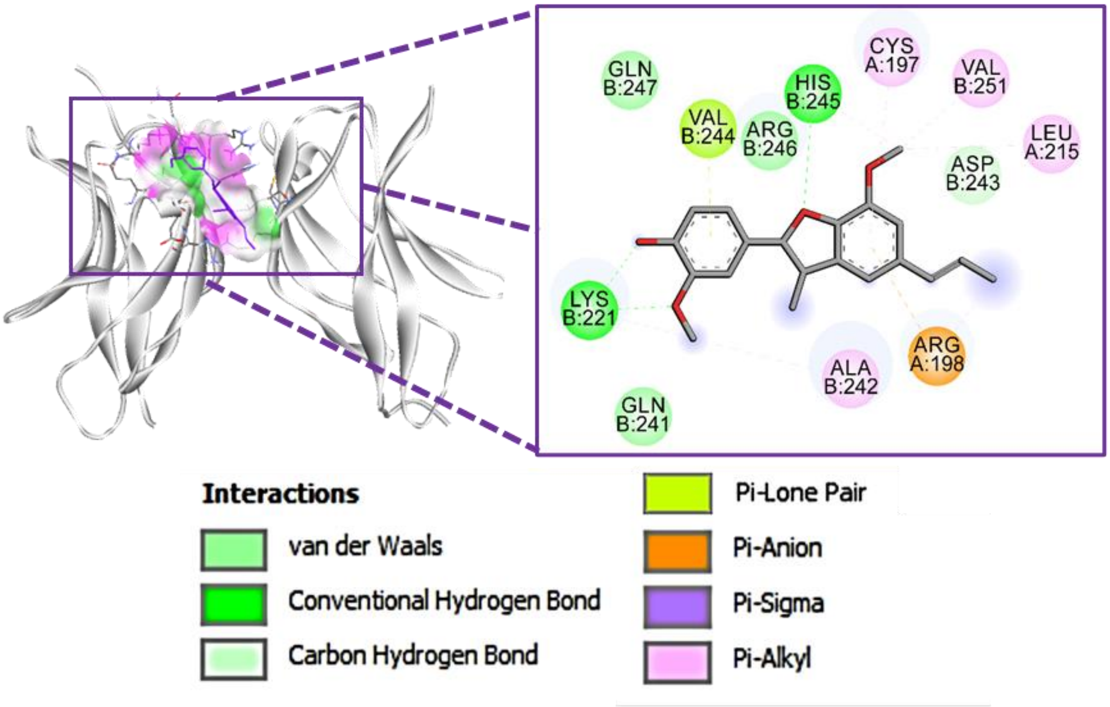

Supplementary Figure S4. Binding interactions of both stereoisomers of compound **1** with relevant amino acids of NF-κBp65.

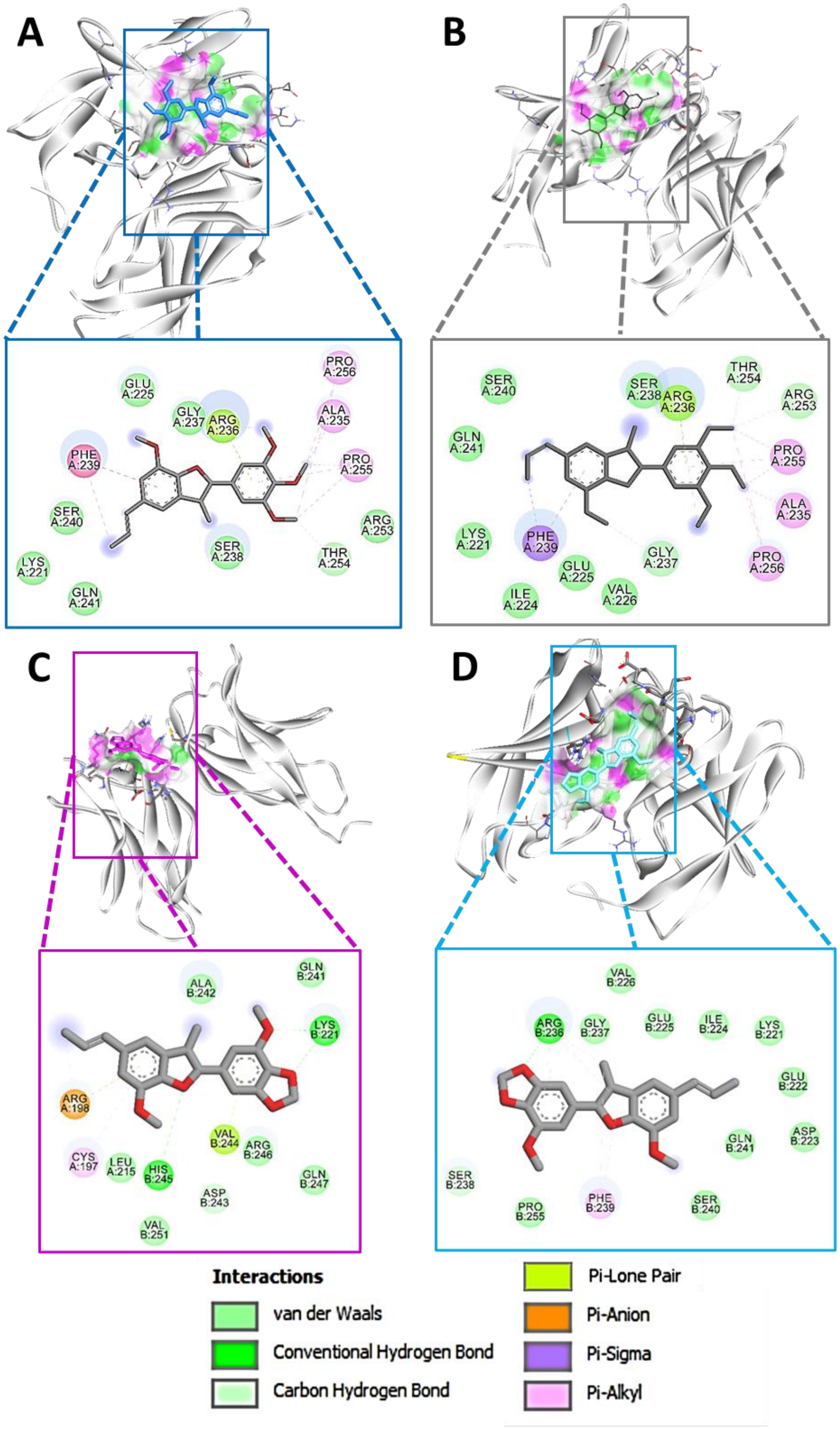

Supplementary Figure S5. Binding interactions of compounds **3**, **4**, **5**, and **6** identified in region A of the molecular network with relevant amino acids of NF-κBp65.

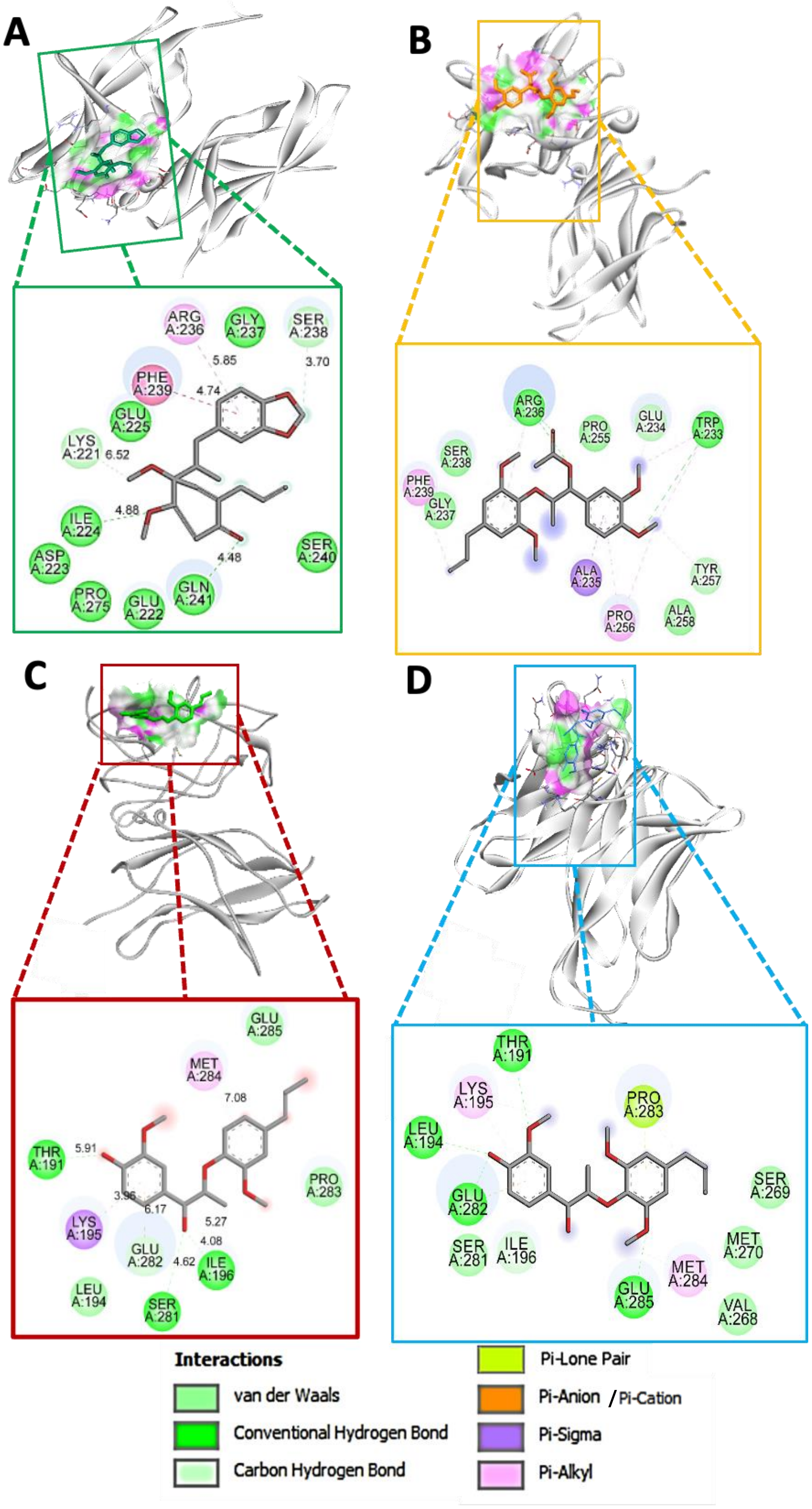

Supplementary Figure S6. Binding interactions of compounds **7**, **8**, **9**, and **10** identified in region B of the molecular network with relevant amino acids of NF-κBp65.

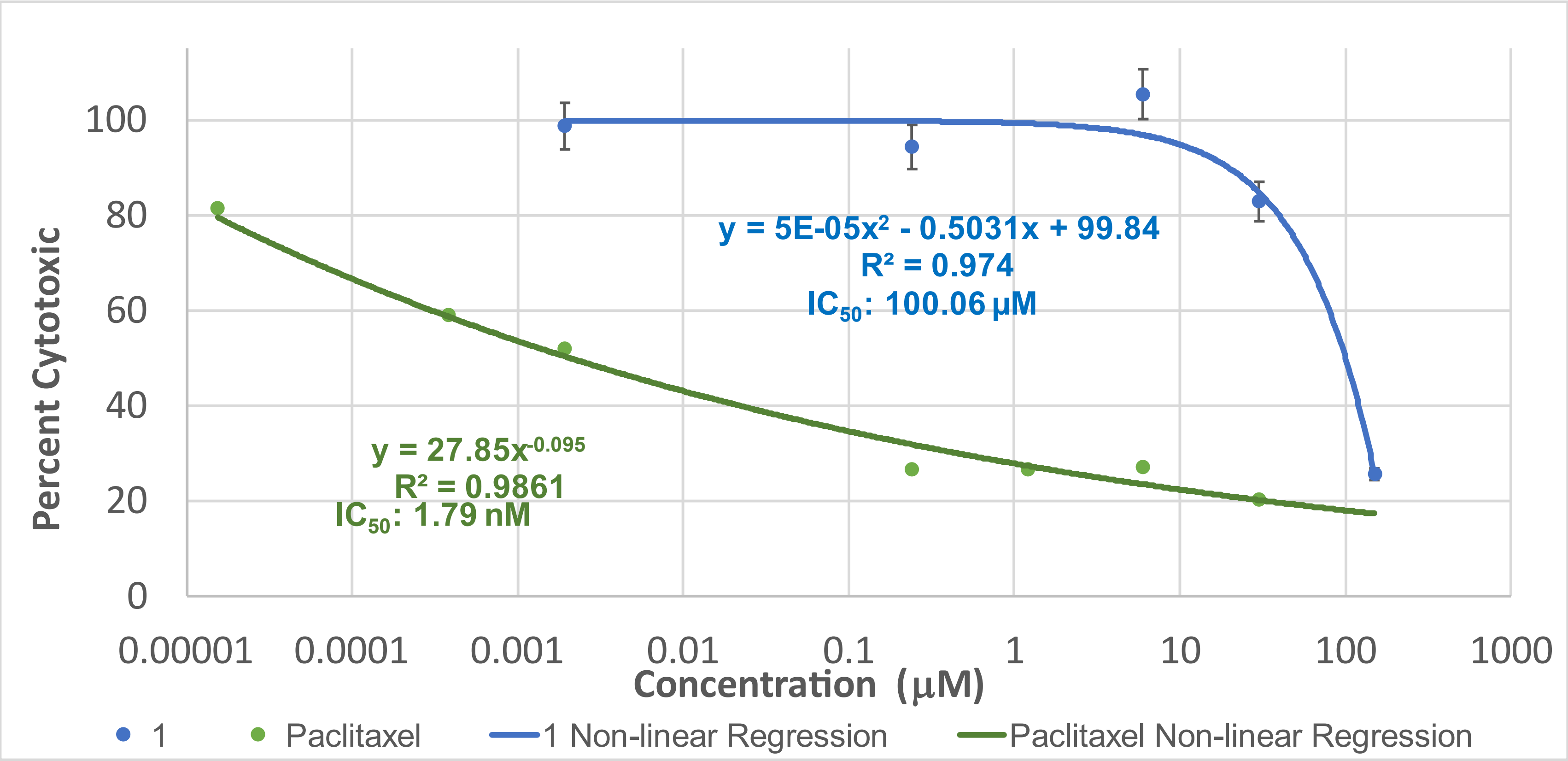

Supplementary Figure S7. Comparative cancer cell cytotoxicity evaluation of licarin A (**1**) and paclitaxel against the DU-145 prostate cancer cell line.
